# Three-dimensional organotypic matrices from alternative collagen sources as pre-clinical models for cell biology

**DOI:** 10.1038/s41598-017-17177-5

**Published:** 2017-12-04

**Authors:** James R. W. Conway, Claire Vennin, Aurélie S. Cazet, David Herrmann, Kendelle J. Murphy, Sean C. Warren, Lena Wullkopf, Alice Boulghourjian, Anaiis Zaratzian, Andrew M. Da Silva, Marina Pajic, Jennifer P. Morton, Thomas R. Cox, Paul Timpson

**Affiliations:** 1 Garvan Institute of Medical Research & The Kinghorn Cancer Centre, Cancer Division, Sydney, NSW 2010 Australia; 2 St Vincent’s Clinical School, Faculty of Medicine, University of NSW, Sydney, NSW 2010 Australia; 3 Beatson Institute of Cancer Research, Switchback Road, Bearsden, Glasgow G61 1BD UK; 4 Institute of Cancer Sciences, University of Glasgow, Glasgow, G61 1QH UK

## Abstract

Organotypic co-cultures bridge the gap between standard two-dimensional culture and mouse models. Such assays increase the fidelity of pre-clinical studies, to better inform lead compound development and address the increasing attrition rates of lead compounds within the pharmaceutical industry, which are often a result of screening in less faithful two-dimensional models. Using large-scale acid-extraction techniques, we demonstrate a step-by-step process to isolate collagen I from commercially available animal byproducts. Using the well-established rat tail tendon collagen as a benchmark, we apply our novel kangaroo tail tendon collagen as an alternative collagen source for our screening-ready three-dimensional organotypic co-culture platform. Both collagen sources showed equal applicability for invasive, proliferative or survival assessment of well-established cancer models and clinically relevant patient-derived cancer cell lines. Additional readouts were also demonstrated when comparing these alternative collagen sources for stromal contributions to stiffness, organization and ultrastructure via atomic force microscopy, second harmonic generation imaging and scanning electron microscopy, among other vital biological readouts, where only minor differences were found between the preparations. Organotypic co-cultures represent an easy, affordable and scalable model to investigate drug responses within a physiologically relevant 3D platform.

## Introduction

Cellular interactions with the extracellular matrix (ECM) occur in a three-dimensional (3D) context and this essential aspect of the tumour microenvironment can lead to altered sensitivity to therapeutics and even act as a barrier to their delivery. This key feature is often overlooked in pre-clinical studies and is likely one of the central factors contributing to the high attrition rates of lead compounds within the pharmaceutical industry, as highlighted recently by our group and others^1,2^. In line with emerging 3D *in vitro* techniques, such as organoid cultures, as pre-clinical testing grounds, there is a need for broader-scale, reproducible and high throughput pre-clinical assays that integrate essential tumour interactions and responses with stromal and ECM components^3–8^.

Collagens are the most abundant ECM component within the body and make up the majority of all interstitial matrix. Despite their abundance, commercially available sources are often expensive and subject to high inter-batch variability, which reduces their reliability for large-scale screening applications. In addition to the costly commercial sources, there are numerous protocols available for either pepsin- or acid-extraction of collagen I from native sources, including the seafood industry, in the form of several fish or cephalopods^9^, or from the more common bovine or pig skin^10–13^. Here, we describe a collagen extraction and organotypic protocol for kangaroo tail, which is based on the acid-extraction technique widely applied to rat tail preparations^14–18^. Rat tail collagen I is the most common source of acid-extracted collagen I, but in the context of large-scale screening, the yield can present a limiting-factor. Conversely, larger kangaroo tails are readily available through online suppliers, which removes the limitation on collagen supply, with the option to keep sizeable stocks for many years and thereby reducing batch-specific variation. Furthermore, work has previously been done using the thicker kangaroo tendons for ligament replacement in medical applications and hence, the optical and histological properties of the fresh fixed tendons are already well defined^19–21^. Here, we detail the step-by-step production of collagen I from kangaroo tail. This novel source is then used to demonstrate the widespread readouts possible using the pre-clinical 3D organotypic matrix platform, employed in parallel with the well-established acid-extracted rat tail collagen I.

This organotypic platform allows assessment of lead compounds in both the stromal compartment or in a 3D co-culture setting. In line with previous work, assessing changes in the stromal compartment^22–25^, we evaluated collagen deposition and remodeling in rat and kangaroo tail matrices by second harmonic generation (SHG) imaging, picrosirius red staining and polarized light microscopy. Similarly, we quantified the matrix stiffness and mechanical properties by atomic force microscopy (AFM) and matrix organization by grey-level co-occurrence matrix (GLCM) analysis. Beyond the previous studies, we also used scanning electron microscopy (SEM) to assess fibre orientation, as a readout of matrix ultrastructure.

Previous work using 3D organotypic co-cultures facilitated assessment of cancer cell clusters by immunofluorescence^26^, allowed correlation of invasion and proliferation using Ki67 staining^27^ and facilitated the association of increased metastasis to increased invasiveness^27,28^. In this work, we demonstrate the invasive capacity of cell lines derived from melanoma, triple-negative breast cancer (TNBC), squamous cell carcinoma (SCC) and pancreatic ductal adenocarcinoma (PDAC). We also highlight the utility of this platform for investigations into the invasive potential of patient-derived cell lines^29^, with an example given from the APGI cohort (Australian Pancreatic Cancer Genome Initiative^30–33^). Finally, we perform a proof-of-principle screen for the effects of two small molecule inhibitors on the invasion and proliferation of a well-established TNBC cell line. The application of kangaroo tail tendon collagen I to generate a novel organotypic matrix demonstrates the wealth of readouts possible from this easily accessible and inexpensive pre-clinical platform.

## Results and Discussion

**Figure 1:**
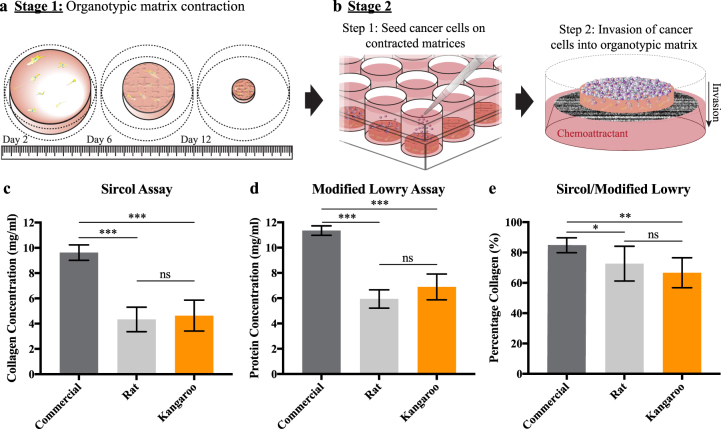
Schematic representation of the three-dimensional (3D) organotypic matrix platform that demonstrates the two stages of the assay and concentration measurements from acid-extraction of collagen from rat and kangaroo tail tendons. (**a**) Stage 1 of the organotypic assay involves contraction of the rat or kangaroo tail collagen by fibroblasts, facilitating stromal-ECM assessment. (**b**) Stage 2 provides opportunities to assess cell invasion, proliferation and survival within a 3D co-culture environment. Application of (**c**) the Sircol^TM^ soluble collagen assay and (**d**) a modified Lowry assay to measure the collagen and total protein concentration of acid-extracted rat and kangaroo tail collagen respectively (n = 7), compared to commercial rat tail collagen (Corning, n = 3). (**e**) Values of collagen and total protein concentration from (**c**,**d**) were used to calculate the collagen abundance in each solution. Mean ± SD.

The organotypic co-culture platform was originally developed as an artificial skin model for the assessment of SCC invasion in a regulated 3D *in vitro* setting^34–36^. Once the pre-clinical power of the model was recognized, the system was further demonstrated for pancreatic cancer^18,23,37–39^ and since, has seen its’ application to the cancer field expanded by us and others^3,4,40–43^. The organotypic matrices applied here have two distinct stages, which are presented as schematics (Fig. 1a,b).

### Assessment of rat and kangaroo tail matrix integrity and structure

The first stage of the organotypic matrix platform involves the generation of a 3D organotypic matrix (Fig. 1a). Here, stromal-ECM assessment can be performed.

#### Isolation of collagen from kangaroo tail

The collagen requirement for larger-scale drug screening using organotypic matrices as a pre-clinical platform can present a significant cost. We propose an alternative source of collagen from kangaroo tail, which can be purchased from online suppliers, at relatively low cost. Based on the detailed anatomical assessment performed by others^21,44^, we successfully isolated tendons from fresh frozen kangaroo tail. A detailed protocol for the acid-extraction is provided in the Supplementary Methods and Supplementary Figure S1. Collagen concentration was not significantly different between rat and kangaroo tail acid-extraction techniques, quantified by a Sircol™ soluble collagen assay (Fig. 1c; commercial rat tail collagen: 9.62 ± 0.61 mg/ml, rat tail collagen: 4.33 ± 0.97 mg/ml, kangaroo tail collagen: 4.64 ± 1.23 mg/ml). This was consistent with the quantification of total protein levels by a modified Lowry assay^45^, which was also not significantly different (Fig. 1d; commercial rat tail collagen: 11.35 ± 0.38 mg/ml, rat tail collagen: 5.94 ± 0.73 mg/ml, kangaroo tail collagen: 6.9 ± 1.02 mg/ml). These two metrics allowed assessment of collagen abundance in the respective collagen preparations, which again showed no significant difference between rat and kangaroo tail acid-extracted collagen preparations (Fig. 1e; commercial rat tail collagen: 84.78 ± 4.93%, rat tail collagen: 66.63 ± 9.87%, kangaroo tail collagen: 72.65 ± 11.44%). However, for both rat and kangaroo tail collagen preparations, the concentration of collagen and protein, and the abundance of collagen were all significantly lower than the commercial rat tail collagen (Corning; Fig. 1c–e).

#### Application of kangaroo tail preparations to fibroblast-driven contraction

**Figure 2:**
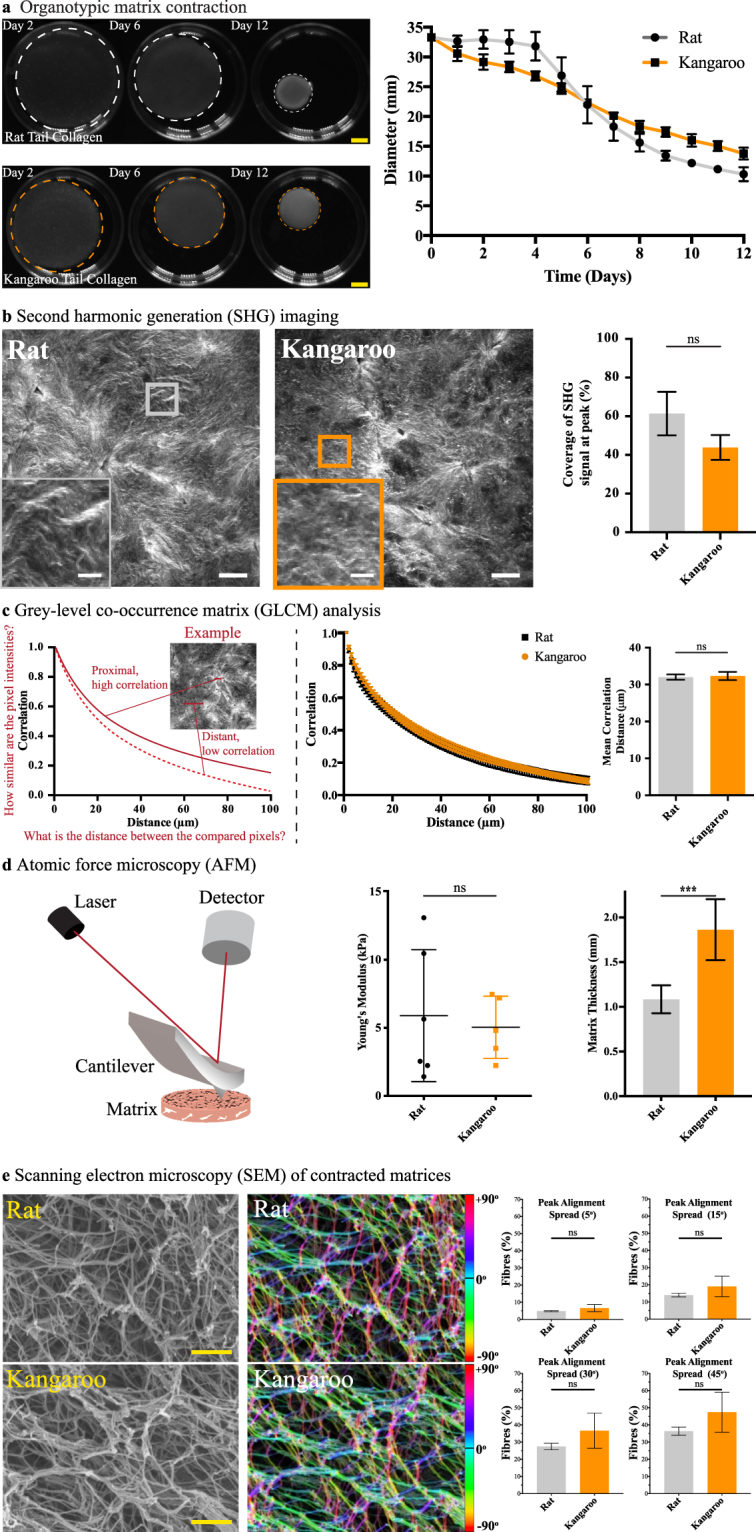
Assessment of rat and kangaroo tail collagen matrix integrity and structure. (**a**) Fibroblast-driven matrix contraction for both rat and kangaroo tail collagens (n = 3, scale bars: 5 mm). (**b**) Contracted matrices were then subjected to second harmonic generation (SHG) imaging of collagen crosslinking (n = 3, scale bars: 50 μm, scale bars (insets): 12.5 μm), (**c**) grey-level co-occurrence matrix (GLCM) analysis of matrix texture (n = 3), (**d**) atomic force microscopy (AFM) of matrix stiffness and thickness by AFM probe engagement (n = 6 (rat) and n = 5 (kangaroo)), and (**e**) scanning electron microscopy (SEM) of contracted rat and kangaroo tail collagen matrices, assessed for changes in fibre orientation angle with the frequency of fibre alignment calculated across different degree ranges spanning the peak alignment (i.e. peak alignment ± 5°, 15°, 30° and 45°; scale bars: 20 μm, n = 3). The local orientation of fibres in the rat and kangaroo tail collagen scanning electron micrographs is represented by the corresponding colour assigned to each specific angle of orientation. Mean ± SD.

The ability of human dermal fibroblasts to contract collagen from rat and kangaroo tail tendons was assessed by establishment of organotypic matrices (Fig. 1a). To generate organotypic matrices, telomerase-immortalized fibroblasts (TIFs)^46^ were embedded in a neutralized collagen matrix of either rat (2.5 ml) or kangaroo (5 ml) tail collagen (~1 × 10^5^ TIFs/matrix), allowing them to contract this matrix over 12 days at 37 °C. This volume was initially optimized to identify the ideal volume of kangaroo tail collagen to reproduce similar matrix characteristics to the benchmarked rat tail collagen matrices (Supp. Fig. S2). By this method, kangaroo tail collagen produced a similar matrix diameter to the well-established rat tail organotypics (Fig. 2a)^23,26,27,42,43,47,48^. These organotypic matrices were then subjected to rigorous assessment of their ECM properties. One common assessment of ECM structure is by intrinsic multiphoton excitation of the non-centrosymmetric structure of cross-linked collagen fibres by SHG imaging^49–52^, which enables assessment of cancer therapies aimed at stromal targeting of the ECM^23,53,54^. Kangaroo tail matrices showed no significant difference in their peak SHG signal (Fig. 2b), indicating that dermal fibroblast remodelling of the collagen is maintained between the preparations. Another readout possible from SHG data allows assessment of matrix order by GLCM analysis (Fig. 2c). GLCM is a mathematical pattern analysis technique that compares the brightness, or grey-level, of each pixel to neighboring pixels (Fig. 2c)^22,55^. In this way, the network of collagen fibres within the matrix can be assessed^22–24,53,54^ and again, no significant difference was found between the rat and kangaroo tail preparations (Fig. 2c). Complementing this, we then applied AFM, a common test of matrix integrity that uses a nanometre tip attached to a cantilever, where changes in the position of the tip are detected by an optical deflection system and provide nanometer resolution for force mapping of samples^53,56–59^. Using AFM, we found no significant difference in the Young’s modulus between the rat and kangaroo tail matrices (Fig. 2d)^60^. Taken together, these orthogonal techniques found no significant differences between the rat and kangaroo tail organotypic matrix preparations (Fig. 2b–d), supporting the use of this novel source of collagen I.

While a larger volume of collagen is required to produce individual kangaroo tail organotypic matrices (see Methods and Supp. Fig. S2), these matrices reach a similar endpoint diameter to the rat tail matrices (Fig. 2a). The increased volume of collagen used to generate kangaroo tail organotypic matrices of suitable diameter was found to result in an increased matrix thickness. To accurately measure this difference, we used the recordings of the z-position of the AFM probe and found that the kangaroo tail organotypic matrices were almost twice as thick as the rat tail matrices (Fig. 2d; rat tail collagen: 1.08 ± 0.16 mm, kangaroo tail collagen: 1.86 ± 0.34 mm). This prompted further analysis by SEM of the ultrastructure of the collagen fibre network, by computational assessment of the alignment of pixels making up collagen fibres across an entire image^61,62^. The peak alignment (measured in degrees) of fibres was determined, and the frequency of fibre alignment calculated across a degree range spanning the peak alignment (i.e. peak alignment ± 5°, 15°, 30° or 45°; Fig. 2e). By this method, we found no significant differences in rat and kangaroo tail collagen fibre alignment frequency (Fig. 2e).

**Figure 3:**
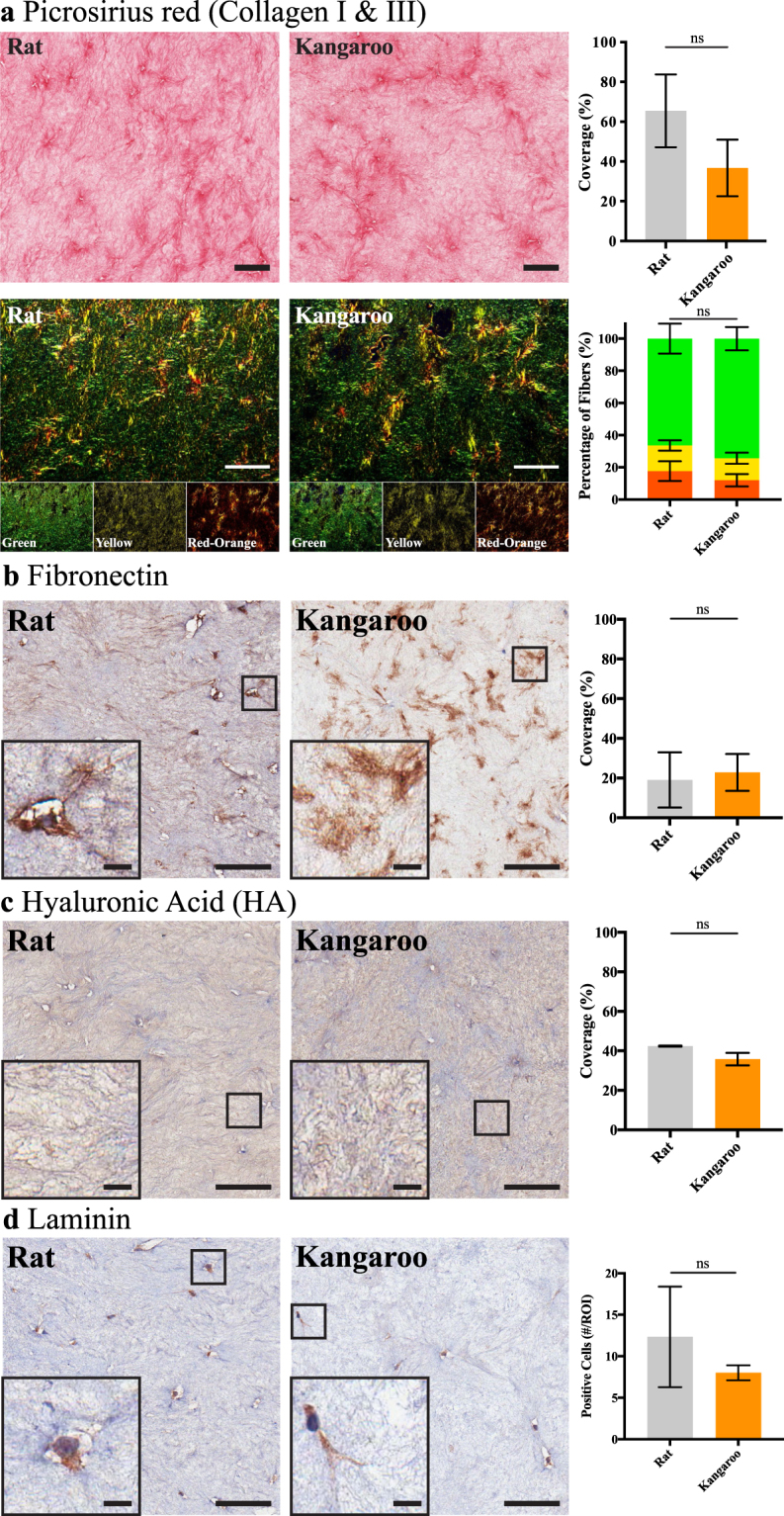
Immunohistochemical (IHC) staining of 12-day fibroblast contracted rat and kangaroo tail collagen matrices for various matrix components. (**a**) Picrosirius red staining (n = 3, scale bars: 100 μm) of collagen content (top, transmitted light imaging) or birefringence (bottom, polarized light imaging; green (low birefringence), yellow (medium birefringence) and red-orange (high birefringence), channels are highlighted for clarity). IHC staining of (**b**) Fibronectin and (**c**) Hyaluronic Acid (HA), quantified for positive staining coverage (n = 3, scale bars: 100 μm, scale bars (insets): 10 μm). (**d**) IHC staining of embedded fibroblasts for Laminin expression, with scoring of positive cells per 500 × 500 μm region of interest (#/ROI; n = 3, scale bars: 100 μm, scale bars (insets): 10 μm). Mean ± SD.

Further to the optical assessment, histological sections were taken for staining of four common ECM components; collagen types I and III, Fibronectin, Hyaluronic Acid (HA) and Laminin (Fig. 3). These ECM components have been shown to have a dual role in cancer, both in containment and progression^63–67^. Picrosirius red staining is a common method to identify collagens in tissue sections and has been previously applied to organotypic matrix sections^23^ and kangaroo articular cartilage and tendons^19,68^. In addition to transmitted light analysis, we applied polarized light microscopy to assess the birefringence of the picrosirius dye molecules, which allows differentiation between highly organized fibrillar collagen and less organized, globular collagen^69,70^. Following staining, no significant change was detected between either the coverage of the picrosirius red stained collagen (transmitted light, Fig. 3a) or the proportions of higher ordered fibrillar collagens (polarized light, Fig. 3a). Immunohistochemical (IHC) staining for Fibronectin was also performed to assess coverage within organotypic matrices and showed no significant difference between the rat and kangaroo tail preparations (Fig. 3b). Similarly, IHC staining for HA, an important stromal-derived ECM component^40,41,71,72^, showed no significant difference in coverage between the rat and kangaroo tail organotypic matrix sections (Fig. 3c). Lastly, strong positive IHC staining of the embedded fibroblasts was observed for Laminin, one of the primary components of the basement membrane (Fig. 3d)^73,74^. Scoring of cells positively stained for Laminin in 500 × 500 μm regions of interest showed no significant difference between the rat and kangaroo tail preparations (Fig. 3d).

**Figure 4:**
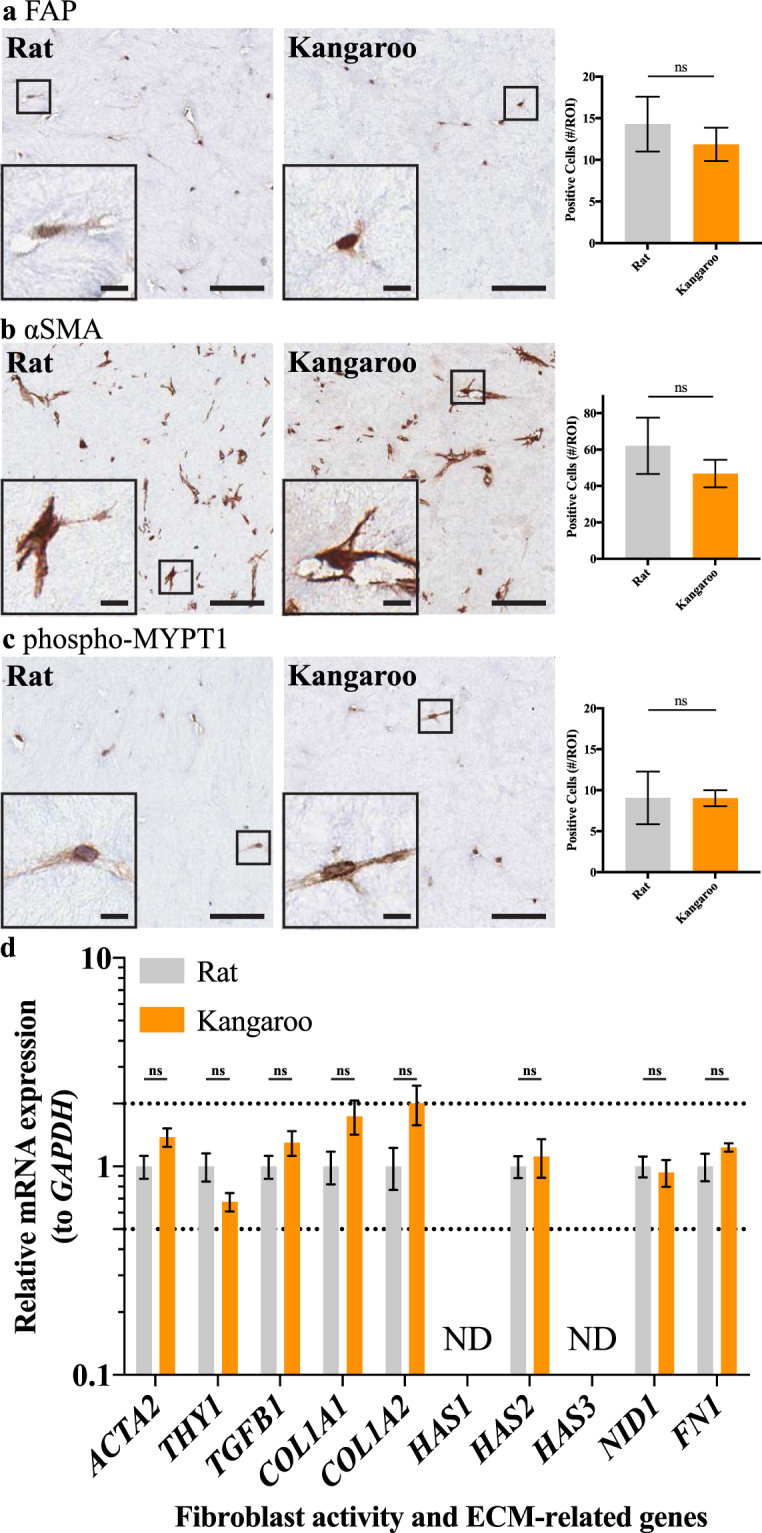
Immunohistochemical (IHC) staining of embedded fibroblasts for three key stromal markers, (**a**) fibroblast activation protein (FAP), (**b**) alpha smooth muscle actin (αSMA) and (**c**) phosphorylated myosin phosphatase target subunit 1 (phospho-MYPT1), scored for positive cells per 500 × 500 μm region of interest (#/ROI; n = 3, scale bars: 50 μm, scale bars (insets): 10 μm). Mean ± SD. (**d**) Quantitative real-time PCR (qRT-PCR) analysis of relative mRNA expression, normalized to *GAPDH*, of genes indicative of fibroblast activity and matrix deposition (n = 4). ND – not detected. Mean ± SEM.

This fibroblast-specific staining for Laminin prompted assessment of the stromal cells themselves (Fig. 4). The embedded fibroblasts are responsible for remodeling the collagen matrices and staining for key markers can inform on their respective activity^67,75,76^. Here, we quantified positive staining by counting cells in 500 × 500 μm regions of interest for all three markers assessed in both rat and kangaroo organotypic matrices; fibroblast activation protein (FAP), a marker of reactive stromal fibroblasts^75,77,78^, alpha smooth muscle actin (αSMA), a marker of increased contractility in myofibroblast cells^56,75,76,79,80^ and phosphorylated-Myosin phosphatase target subunit 1 (phospho-MYPT1), a marker of actomyosin contractility^76,81–83^ (Fig. 4a–c). This affirmed that the activity of the stromal cells within both the rat and kangaroo tail matrices was not significantly different between the two preparations. Previous work has demonstrated that activated fibroblasts, which are capable of secreting their own ECM^3,84–87^, also upregulate several genes at the transcriptional level^87^. To confirm that these genes were similarly expressed in organotypic-embedded fibroblasts, within both rat and kangaroo tail organotypic matrices, quantitative real-time PCR (qRT-PCR) was performed on fibroblasts from 12 day contracted organotypic matrices. According to the MIQE guidelines^88^ for the analysis of qRT-PCR data by the comparative CT method^89,90^, a minimum of 2-fold difference is necessary to report differences in gene expression between treatment groups. The changes presented for the fibroblast markers *ACTA2* (i.e. αSMA), *THY1* and *TGFB1* are neither statistically significant, nor their fold-change of a significant magnitude, to show a difference between the rat and kangaroo tail matrices (Fig. 4d). These genes have previously been shown to be upregulated in activated fibroblasts, along with *COL1A1, COL1A2* and *FN1* ^87^. Here we saw no significant difference in these additional transcripts between rat and kangaroo tail matrices, even with the additional assessment of the hyaluronan synthesis genes *HAS1, HAS2* and *HAS3*, and the basement membrane glycoprotein, Nidogen-1 (*NID-1*; Fig. 4d). This suggests that there is no significant difference in activation status of fibroblasts embedded in the two different matrices. Additionally, the *GAPDH* normalized results presented (Fig. 4d) were consistent with normalization to a second housekeeping gene *RPLP0* (Supp. Fig. S3).

Targeting of the tumour stromal-ECM components is an emerging area, as their respective importance in both impeding drug delivery and therapeutic resistance are increasingly recognized^23,25,71,72,79,91–98^. Using stage 1 (Fig. 1a) of our novel kangaroo tail organotypic matrix platform, which demonstrated similar properties to the well-defined rat tail organotypic matrices, could allow assessment of this stromal targeting on both the ECM and the associated stromal cells, as demonstrated previously^23–25,47,53^. Subsequent assessment of cancer cell behavior, either independently or in parallel with stromal-ECM modulation, is then possible by progressing to stage 2 of the organotypic assay (Fig. 1b).

### Application of kangaroo tail collagen organotypic matrices to several common cancer models and patient-derived cell lines

**Figure 5:**
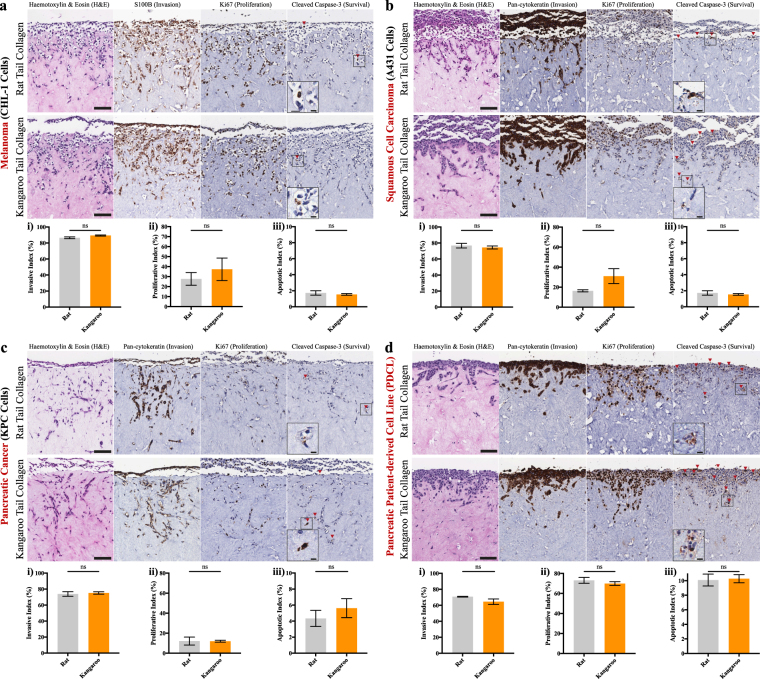
Organotypic invasion assays demonstrating the multiple readouts possible from several established cancer cell lines and an example patient-derived cell line (PDCL) model. Representative images and quantification are given for (**a**) melanoma (CHL-1, n = 3), (**b**) squamous cell carcinoma (SCC; A431, n = 3), (**c**) pancreatic ductal adenocarcinoma (PDAC; KPC, n = 3) and (**d**) a PDAC PDCL (n = 3). Each cell line was then scored for invasive cells (i, S100B/pan-cytokeratin, which excludes fibroblasts), proliferating cells (ii, Ki67) and apoptotic cells (iii, cleaved caspase-3). Scale bars: 100 μm. Scale bars (insets): 12.5 μm. Mean ± SEM.

Organotypic matrices were originally developed as models of artificial skin for modeling invasion of SCC^34–36^. The method itself involves the initial generation of an organotypic matrix (Fig. 1a); here using rat and kangaroo tail collagen as the substrate for TIFs. After 12 days, the matrix is sufficiently remodeled to allow seeding of cancer cells on the upper surface (Fig. 1b, 1 × 10^5^ cancer cells per matrix). Once the seeded cells have grown to confluence, generally after 5 days, the seeded matrices are moved to an air-liquid interface to allow the seeded cells to invade towards the chemoattractants in the growth media (Fig. 1b)^18,34–36^. In this work, we demonstrate invasion of cell lines from human melanoma (CHL-1, Fig. 5a), human SCC (A431, Fig. 5b) and mouse PDAC (KPC, Fig. 5c) that invaded on an air-liquid interface for 14 days; recapitulating previous work using rat tail organotypic matrices^40,41,43,99,100^. From here, we demonstrate key functional readouts possible from IHC staining, such as cell invasion using S100B/pan-cytokeratin expression, which excludes fibroblasts from the quantification, proliferation by Ki67 and survival by cleaved caspase-3. Invasion scoring was performed by first counting the cell layer adjacent to the top of the collagen matrix (non-invaded cells) and adding this value to the total number of cells within the matrix, to generate a percentage of invaded cells, or the “invasive index” (Fig. 5). This is similar to the scoring performed for proliferation or survival, where the cells that are positively stained for Ki67 or cleaved caspase-3 respectively (brown) are divided by the total number of cells (brown + blue), to generate proliferative and apoptotic indices (Fig. 5). Notably, Fig. 5 shows that for each individual cancer cell line, invasion, proliferation and survival were demonstrably similar between rat and kangaroo tail matrices, while illustrating the clear differences in 3D invasion, proliferation and survival between different cancer types. Importantly, we also demonstrate the translational applicability of our novel organotypic matrix platform by assessment of a patient-derived cell line (PDCL) from PDAC (Fig. 5d)^23,33^. Patient-derived xenografts and PDCL models are gaining momentum in the field, as they maintain the heterogeneity of patient tumours, facilitating more accurate pre-clinical drug screening^29,98^ and can be easily applied to our pre-clinical 3D organotypic platform (Fig. 5d).

**Figure 6:**
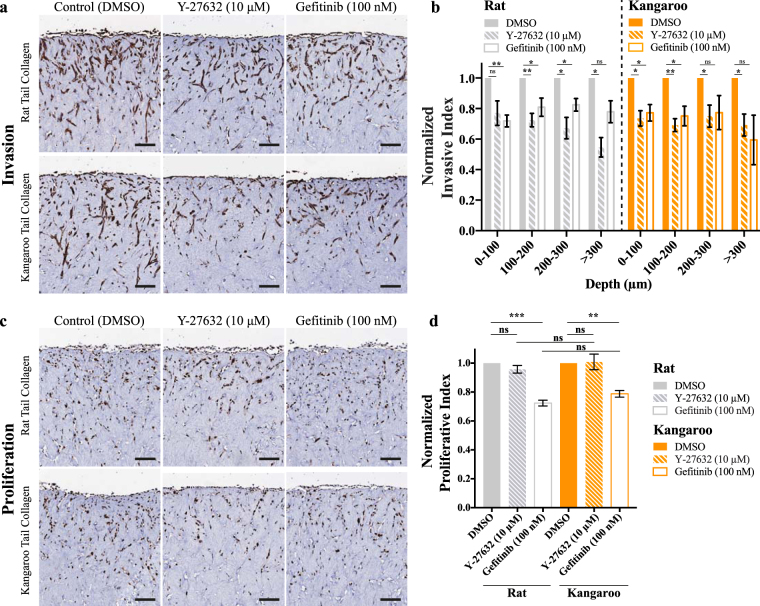
An example of a small-scale pre-clinical drug screen with the TNBC breast cancer cell line MDA-MB-231, invading into organotypic matrices and treated for 7 days with either control (DMSO), Y-27632 (10 μM) or Gefitinib (100 nM). Representative images are given for (**a**) pan-cytokeratin staining, which was (**b**) scored for invasion depth (n = 4). Representative images are also given for (**c**) Ki67 staining, which was (**d**) scored for proliferation (n = 4). Scale bars: 100 μm. Scale bars (insets): 12.5 μm. Mean ± SEM.

Finally, to confirm the pre-clinical applicability of the rat and kangaroo tail organotypic matrices, we performed a proof-of-principle drug screen using the well-established human MDA-MB-231 TNBC cell line (Fig. 6). TNBCs are currently the only subtype of breast cancer without approved targeted therapies^101^ and thus, the use of physiologically relevant 3D models for therapeutic development could be critical to developing novel targets and therapies. It has previously been shown that ROCK inhibition by Y-27632 can inhibit the infiltration of MDA-MB-231 cells using 2D *in vitro* assays^102–104^. Conversely, the EGFR inhibitor Gefitinib has been shown to have potent anti-proliferative effects on MDA-MB-231 cells^105–107^, while the anti-invasive effect is less well established^108–110^. To quantify the changes in invasion depth upon inhibition, invaded MDA-MB-231 cells at each 100 μm increment were divided by the number of cells on the surface of the collagen matrix, in a similar manner to the invasive index calculated in Fig. 5. These values were then normalized to the vehicle (DMSO) control at each depth and compared between treatments. Here, we incubated the MDA-MB-231 cells on an air-liquid interface for 7 days, then treated with either vehicle (DMSO) control, Y-27632 (10 μM) or Gefitinib (100 nM) and incubated for a further 7 days. By this approach, we confirm that inhibition of ROCK by Y-27632 reduces the 3D invasive ability of these cells (Fig. 6a,b). In this setting, we saw no change in proliferation following Y-27632 treatment (Fig. 6c,d) in either rat or kangaroo matrices. In contrast, the EGFR inhibitor Gefitinib, showed reductions in both the invasion and proliferation of the MDA-MB-231 cells, as they invaded into the 3D rat and kangaroo matrices (Fig. 6a–d), supporting the effect shown for other cancers^111–113^. Our data not only support the current canon, but also provide evidence as to the power of this 3D organotypic matrix platform for future pre-clinical drug screening studies, targeting either the stromal-ECM compartment or the cancer cells themselves.

## Conclusions

In this work, we present a systematic comparison between the use of acid-extracted collagen I from rat and kangaroo tail tendons. This acts as a proof-of-concept for novel alternative collagen sources to be applied to the 3D pre-clinical organotypic matrix platform (Fig. 1a,b). We demonstrate assays aimed at monitoring perturbations in the ECM and the parallel effects on the stromal cells, responsible for the matrix contraction. Further to this, the invasion of four well-established cancer cell lines was demonstrated, and we highlight the use of PDCLs for future pre-clinical investigations. The power of this assay was then underlined using a small-scale proof-of-principle drug screen, confirming the anti-invasive effect of Y-27632 and establishing a dual role for Gefitinib in both inhibition of invasion and proliferation in a TNBC cell line. Further development of the assay could include the use of transgenic, patient-matched or cancer-associated fibroblasts, which will allow specific questions to be asked about the stromal interactions with the ECM and the co-cultured cancer cells^23,114,115^. Similarly, keratinocytes have also been cultured above the contracted matrices from stage 1 (Fig. 1a), allowing assessment of the regenerative ability of skin upon various perturbations^116,117^. As cheaper and more convenient sources of collagen I become available, the pre-clinical applicability and wide variety of readouts possible from this high fidelity 3D assay will pave the way to its’ widespread integration into translational research in the future.

## Methods

### Atomic Force Microscopy (AFM)

As described previously^23^, measurement of the matrix Young’s modulus was performed on a Bioscope Catalyst (Bruker) mounted on a TMC anti-vibration table (Technical Manufacturing Corporation), using a 1 μm spherical colloidal probe (spring constant = 0.06 N/m; Novascan). First, the probe was calibrated on an uncoated glass substrate, by measuring the deflection sensitivity of the probe in fluid and upon engagement. Prior to matrix assessment, organotypics were immobilized in a 40 mm glass bottom cell culture dish using a 10% agarose solution (Bioline). To determine the spring constant, a thermal tune sweep was performed. Indentation assessment was run on 3 different areas/matrix and 9 points/area (separated by 35 μm) using a Peak Force Tapping mode, with an average loading force of 1 nN, prior to calculating Young’s modulus values from force curves by the Hertz spherical indentation model (AtomicJ)^60^. Matrix thickness was also calculated for each area by subtracting the z position of the point of contact between the probe and the matrix, and the z position of the glass bottom cell culture dish.

### Cell culture and reagents

The *LSL-KRas* ^*G12D/*+^ *, LSL-Trp53* ^*R172H/*+^ *, Pdx1-Cre* (KPC) PDAC^100^, CHL-1, A431 and telomerase-immortalized fibroblast (TIF)^46^ cell lines were maintained in Dulbecco’s modified Eagle medium (DMEM; Gibco), while MDA-MB-231s were maintained in Roswell Park Memorial Institute (RPMI) 1640 (Gibco), both were supplemented with 10% FBS and penicillin/streptomycin at 100 U/ml. The PDAC patient-derived cell line (PDCL) TKCC10 was maintained in an m199/Ham F12 media mixture, described in^118^. Commercial rat tail collagen was from Corning (354249). Y-27632 (Selleckchem, S1049) and Gefitinib (Cayman Chemicals, 13166) were made up as 10 mM stock solutions in DMSO.

### Grey-level co-occurrence matrix (GLCM) analysis

To assess stromal collagen fibre organization and crosslinking, GLCM analysis was used to characterize the texture of the organotypic matrix samples by determining the correlation of the SHG signal intensity within the matrix, as a function of distance, where a slower decay shows a more organized and correlated network of collagen fibres^22,53,55^. SHG images were acquired with the laser power adjusted to give an approximately uniform intensity between images. For each matrix within a triplicate, 5 representative regions were taken with a field-of-view of 512 × 512 μm, line averaging of 32 and a scan speed of 400 Hz. GLCM analysis was performed using a custom Matlab script, available at https://github.com/timpsonlab/shg-quantification-tools. The average GLCM texture parameters^119^ were calculated between pixel offsets in 1 pixel increments, up to 100 pixels, at 0°, 90°, 180° and 270° orientations. The image correlation, as a function of distance, was plotted and the mean correlation distance ${\rm{D}}=\,\frac{{\sum }_{{\rm{i}}}{{\rm{d}}}_{{\rm{i}}}{\rm{c}}({{\rm{d}}}_{{\rm{i}}})}{{\sum }_{{\rm{i}}}{\rm{c}}({{\rm{d}}}_{{\rm{i}}})}$, where d_i_ is the offset of the i^th^ pixel and c(d) is the GLCM correlation, as a function of distance.

### Immunohistochemistry (IHC)

Organotypic matrices were fixed in 10% neutral buffered formalin, prior to paraffin block embedding. 4 μm sections were then either haematoxylin and eosin (H&E) stained on a Leica Autostainer or underwent IHC staining for cleaved caspase-3(Asp175) (Cell Signaling, 9661, 1:100), Ki67(SP6) (ThermoFisher Scientific, RM-9106-S1, 1:500), S100B (Dako, Z0311, 1:3000), pan-cytokeratin (Leica-Novocastra, NCL-C11, 1:50), Fibronectin (EP5; Santa Cruz Biotechnology, sc-8422, 1:100) or pan-Laminin (Abcam, ab11575, 1:100). A detailed procedure for the above IHC on a Leica Bond RX is provided in the Supplementary Methods.

For the following antibodies, manual IHC staining was performed using a pH6 target retrieval solution (Dako, S1699) at 93 °C for 30 minutes, prior to a 10 minute cool down in running water, followed by to a 5 minute peroxide block (Dako, K4011) and 10 minute protein block (Dako, X0909), then overnight incubation at 4 °C with one of the below primary antibodies pMYPT1(Thr696) (Millipore, ABS45, 1:100), FAP (Abcam, 53066, 1:500) or αSMA (Abcam Australia, AB5694, 1:200). Detection was then performed using the EnVision+ System HRP Labeled Polymer (Dako, Anti-Rabbit, K4001) and DAB for 10 minutes. For detection of Hyaluronic Acid (HA), the additional use of a Biotin and Avidin Blocking System (Dako, X0590) was required, prior to a 60 minute incubation with hyaluronic acid binding protein (Calbiochem, 385911, 1:300) and detection using the VECTASTAIN Elite ABC HRP kit (Vector Labs, PK-6100) and DAB for 10 minutes. All images were taken using an Aperio CS2 ScanScope (Leica Biosystems). For quantification of positively staining of HA and Fibronectin in ImageJ (NIH), colour deconvolution was used to isolate the DAB staining prior to measurement of staining coverage. Scoring of pMYPT1, αSMA, FAP and Laminin staining of fibroblasts within organotypic matrix sections was performed in 10 representative 500 × 500 μm regions for each biological replicate.

### Organotypic assay

Rat or kangaroo tail tendon collagen was prepared by acid-extraction with 0.5 M acetic acid (see Supplementary Methods). Concentration of these collagen preparations was quantified by a Sircol^TM^ soluble collagen assay, as per the manufacturer’s protocol, and a modified Lowry assay, described in^45^. The method for organotypic matrix production has been described previously for rat tail collagen^15,18^. Using the rat tail collagen matrices as a benchmark, we optimized the kangaroo tail collagen preparation volume to produce a matrix of similar collagen density and mechanical properties (Supp. Fig. S2). Briefly, collagen I matrices were prepared with acid-extracted collagen, 10X MEM (Gibco) and neutralized using sodium hydroxide, prior to addition of FBS containing ~1 × 10^5^ TIFs/matrix. To maintain consistent pore sizes throughout the work, polymerization was allowed to occur at 37 °C^12,120,121^. Detached polymerized matrices (2.5 ml or 5 ml) in 6-well wells were allowed to contract for 12 days. For organotypic invasion assays, contracted matrices were subsequently seeded with 1 × 10^5^ cancer cells in complete media, which were allowed to grow to confluence over 5 days. Seeded matrices were then mounted on metal grids, raising to an air/liquid interface, which was fed from below by complete media; changed every 2 days. Matrices were fixed after 14 days (MDA-MB-231, A431, KPC and CHL-1 cells) or 21 days (TKCC10 cells) of invasion. For scoring, 9 representative 500 × 500 μm areas were selected from each condition and replicate. Invasive cells were identified by pan-cytokeratin or S100B staining and recorded as having invaded 0–100 μm, 100–200 μm and 200–300 μm and > 300 μm. The invasive index was then calculated as either the number of invaded cells, divided by the total number of cells (sum of invaded cells and cells on the surface of the matrix) or by calculating a ratio of invaded cells at each depth to the cells on the surface. The proliferative and apoptotic indices were taken as a ratio of Ki67 or cleaved caspase-3 positive cells respectively, divided by the total cell number of cells in each area.

### Picrosirius red staining and quantification

4 μm sections were taken from paraffin embedded organotypic matrices and underwent deparaffinisation, rehydration and staining with 0.1% picrosirius red (Polysciences), as per the manufacturer’s protocol. Collagen coverage was then quantified using an in-house ImageJ macro (n = 3 matrices, 20 regions/matrix)^23^. Polarized light images were collected using an Olympus U-POT polarizer in combination with an Olympus U-ANT transmitted light analyser fitted to the microscope. Automated quantitative intensity measurements of fibrillar collagen birefringent signal were carried out on polarized light images using ImageJ as previously described^23^. Briefly, for each polarized light image, Hue-Saturation-Balance (HSB) thresholding was applied, where 0 ≥ H ≤ 29 | 0 ≥ S ≤ 255 | 70 ≥ B ≤ 255 was used for red-orange (highly birefringent) fibres, 30 ≥ H ≤ 44 | 0 ≥ S ≤ 255 | 70 ≥ B ≤ 255 for yellow (medium birefringent) fibres and 45 ≥ H ≤ 245 | 0 ≥ S ≤ 255 | 70 ≥ B ≤ 200 for green (low birefringent) fibres. The relative area (as a % of total fibres [0 ≥ H ≤ 245 | 0 ≥ S ≤ 255 | 70 ≥ B ≤ 200]) was then calculated.

### RNA isolation, reverse transcription and quantitative real-time PCR (qRT-PCR) experiments and analysis

Prior to RNA isolation, rat and kangaroo organotypic matrices were prepared as described above and after contracting for 12 days, were directly collected into QIAzol lysis reagent (Qiagen) in Lysing Matrix S tubes (MP Biomedicals). The matrices were then disrupted using the FastPrep-24™ 5 G Homogenizer (MP Biomedicals). RNA samples were isolated using the miRNeasy kit (Qiagen) and reverse transcribed with the Transcriptor First Strand cDNA Synthesis Kit (Roche Diagnostics). cDNA was synthesized from 2 μg of total RNA and diluted 1:10 before any further analysis. qRT-PCR experiments were performed using the Roche Universal Probe Library System on a Roche LightCycler480^®^ (Roche LifeScience). Probes and programs used for qRT-PCR analysis are listed in Supplementary Tables S1 and S2. Relative mRNA expression levels were normalized to *GAPDH* or *RPLP0*, and quantification was performed using the comparative CT method described previously^89,90^, for each biological replicate. Relative expression in organotypic-embedded fibroblasts within kangaroo tail matrices was compared to its expression within the rat tail organotypic matrices, referred as 1. SEM of the **ΔΔ**Ct values was calculated according to the fold change.

### Scanning Electron Microscopy (SEM) and fibre orientation analysis

Contracted collagen matrices were fixed with 2% glutaraldehyde, prior to step-wise dehydration in ethanol of increasing concentration (30–100%). Samples were then subjected to CO_2_ critical point drying, spattered with gold and scanned on a Hitachi S3400 with an accelerating voltage of 15 kV. Fibre orientation analysis was performed on electron micrographs using an in-house ImageJ (NIH) macro, as previously described^61,62^. Briefly, structure tensors were derived from the local orientation and isotropic properties of pixels that make up collagen fibrils. Within each input image, these tensors were evaluated for each pixel by computing the continuous spatial derivatives in the x and y dimensions using a cubic B-spline interpolation. From this, the local predominant orientation was obtained. The peak alignment (measured in degrees) of fibres was then determined, and the frequency of fibre alignment calculated across different degree ranges spanning the peak alignment (i.e. peak alignment ± 5°, 15°, 30° and 45°).

### Second Harmonic Generation (SHG) imaging and analysis

SHG imaging was performed on 10% neutral buffered formalin fixed samples on an inverted Leica DMI 6000 SP8 confocal microscope with a 25X water objective. Multiphoton excitation was performed at 890 nm with a Ti:Sapphire femtosecond laser (Coherent Chameleon Ultra II), detecting SHG intensity with an RLD HyD detector (440/20 nm). For each matrix within a triplicate, 3 representative 80 μm z-stacks were taken with a field-of-view of 512 × 512 μm, a line averaging of 4, a scan speed of 400 Hz and a z-step size 2.52 μm. The coverage of the SHG signal was measured using ImageJ (NIH) across the z-stacks and the peak signal was used for comparisons between rat and kangaroo tail organotypic matrices. Representative SHG images were taken from the peak intensity of the z-stack, with a field-of-view of 512 × 512 μm, a line averaging of 32 and a scan speed of 400 Hz.

### Statistical analysis

Data were statistically analyzed in GraphPad Prism (GraphPad Software, Inc., CA) with Student’s t-tests. For normalized data, a one-sample t-test was performed against the normalized value. In all cases, statistical significance was given as *p < 0.05, **p < 0.01 and ***p < 0.001.

## Electronic supplementary material

Supplementary Information
