# Supplementary material for: Three-dimensional organotypic matrices from alternative collagen sources as pre-clinical models for cell biology
Source: Sci Rep. 2017 Dec 4;7:16887. doi: 10.1038/s41598-017-17177-5 (PMC5715059; doi:10.1038/s41598-017-17177-5)
Supplement: Supplementary file 1 — Supplementary Information [file 41598_2017_17177_MOESM1_ESM.pdf]

Three-dimensional organotypic matrices from alternative collagen sources as pre-clinical models for cell biology

James R.W. Conway[1], Claire Vennin[1], Aurélie S. Cazet[1], David Herrmann[1], Kendelle J. Murphy[1], Sean C. Warren[1], Lena Wullkopf[1], Alice Boulghourjian[1], Anaiis Zaratzian[1], Andrew M. Da Silva[1], Marina Pajic[1], Jennifer P. Morton[2], Thomas R. Cox*[1] and Paul Timpson*[1]

[1]Garvan Institute of Medical Research & The Kinghorn Cancer Centre, Cancer Division, Sydney, NSW 2010, Australia; St Vincent's Clinical School, Faculty of Medicine, University of NSW, Sydney, NSW 2010, Australia
[2]Beatson Institute of Cancer Research, Switchback Road, Bearsden, Glasgow G61 1BD, UK
* Correspondence to Dr. Paul Timpson: p.timpson@garvan.org.au and Dr. Thomas R. Cox: t.cox@garvan.org.au.

Supplementary Methods

Preparation of collagen I from kangaroo tail (Supp. Fig. S1)

Protocol is for 1 kg fresh or frozen kangaroo tail.

Day 1

1. Remove tendon as follows:
   - Grip tendon tightly using three pronged tweezers.
   - Pull free of the epithelium, taking care not to tear.
   - Collect all the tendons in 50 ml tubes. There should be enough to three-quarter fill three 50 ml tubes.
2. Solubilise the collagen I from each 50 ml tube of tendons in a 2 L conical flask filled with 1500 ml of 0.5 M acetic acid (~1 g/250 ml) for ≥48 hours at 4°C. Use a magnetic stirrer to help solubilise.

Day 4

3. Filter the collagen extract through a strainer or colander, to remove the epithelial waste from the tendons.
4. In a 2 L conical flask, slowly add sodium chloride to the supernatant to make a 10% (w/v) solution.
5. The collagen I should form a single opaque solution, no longer separating. Centrifuge at 10,000 rpm at 4°C for 1 hour.
6. Discard supernatant.
7. Re-dissolve precipitate in ~500 ml of 0.25 M acetic acid for 24 hours at 4°C. Volume can differ depending on yield.

Day 5-7

8. Dialyse the collagen solution against 6-8 changes of 5 L of 17.4 mM acetic acid (5 ml glacial acetic acid (17.4 M) into 5 L of distilled water) at 4°C. Use a magnetic stirrer in the bottom of the bucket to increase dialysis efficiency. This is changed twice daily.
9. Centrifuge the dialysed collagen I at 10,000 rpm at 4°C for 1.5 hours.
10. In a biological safety cabinet, decant supernatant into a cold glass bottle. UV light sterilizes the collagen at 4°C for 20 minutes in the cabinet.
11. Adjust the collagen I concentration using 17.4 mM acetic acid in sterile water, at 4°C. Collagen concentration can be measured using a Sircol™ soluble collagen assay and/or a modified Lowry assay[45].

Preparation of collagen I from rat tails

Protocol is for 12-14 fresh or frozen medium-sized rat tails.

Day 1

1. Wash tails in ethanol, leaving to soak and loosen for 10-15 minutes.
2. Remove tendon as follows:
    - Rotate scalpel blade around proximal region of tail.
    - Cut the skin of the tail along its length.
    - Tear away the skin from the proximal cut.
    - Grip mid-way between the largest part of the tendon with toothed forceps.
    - Twist and pull tendon from upper sheath.
    - Tear towards tip, careful not to take too much sheath in the process.
    - After extraction, wash tendons in ethanol and then in distilled water.

    Note: Harvested tendons can be frozen at -80°C for 15 minutes and smashed to increase surface area for the acetic acid to dissolve the collagen I and increase yield.
3. Solubilise collagen I in 1500 ml of 0.5 M acetic acid for ≥48 hours at 4°C. Use a magnetic stirrer to help solubilise.

Day 4

4. Filter the collagen extract through gauze, to remove any remaining sheath from tendon extraction.
5. In a 2 L conical flask, slowly add sodium chloride to the supernatant to make a 10% (w/v) solution.
6. The collagen I should form a single opaque solution, no longer separating. Centrifuge at 10,000 rpm at 4°C for 1 hour.
7. Discard supernatant.
8. Re-dissolve precipitate in ~500 ml of 0.25 M acetic acid for 24 hours at 4°C. Volume can differ depending on yield.

Day 5-7

9. Dialyse the collagen solution against 6-8 changes of 5 L of 17.4 mM acetic acid (5 ml glacial acetic acid (17.4 M) into 5 L of distilled water) at 4°C. Use a magnetic stirrer in the bottom of the bucket to increase dialysis efficiency. This is changed twice daily.
10. Centrifuge the dialysed collagen I at 10,000 rpm at 4°C for 1.5 hours.
11. In a biological safety cabinet, decant supernatant into a cold glass bottle. UV light sterilizes the collagen at 4°C for 20 minutes in the cabinet.
12. Adjust the collagen I concentration using 17.4 mM acetic acid in sterile water, at 4°C. Collagen concentration can be measured using a Sircol™ soluble collagen assay and/or a modified Lowry assay[45].

Immunohistochemistry (IHC) protocol for organotypic matrices

Formalin fixed paraffin embedded (FFPE) organotypic matrix specimens were sectioned at 4 μm using a Leica RM2235 microtome. Sections were placed on a Superfrost plus slide and allowed to incubate at 60°C overnight, to maximise adhesion.

Leica Bond RX Protocol:

Dewax: Bond Dewax Solution (Leica, AR9222).

Heat-Induced Epitope Retrieval (HIER) protocol: HIER 30 min with ER2 (EDTA, pH9) at 93°C. **Except Cleaved Caspase-3**, which is 20 min with ER2 (EDTA, pH9) at 93°C.

Step #	Reagent	Incubation (min)
1	*Peroxide Block	5:00
2	Bond Wash Solution	0:00
3	Bond Wash Solution	0:00
4	Antibody	60:00
5	Bond Wash Solution	0:00
6	Bond Wash Solution	0:00
7	Bond Wash Solution	0:00
8	*Post Primary	15:00
9	Bond Wash Solution	2:00
10	Bond Wash Solution	1:00
11	Bond Wash Solution	0:00
12	*Polymer	15:00
13	Bond Wash Solution	2:00
14	Bond Wash Solution	1:00
15	Bond Wash Solution	0:00
16	*Mixed DAB Refine	0:00
17	*Mixed DAB Refine	10:00
18	Bond Wash Solution	0:00
19	Deionised Water	0:00
20	Deionised Water	0:00
21	Deionised Water	0:00
22	Deionised Water	0:00

*Reagents all part of Leica Bond Polymer Refine Detection (DS9800)

Reagents:

Bond Epitope Retrieval (ER) Solution 2 (EDTA based buffer, pH8.9-9.1) (Leica, AR9640)
Bond Wash Solution 10x concentrate (Leica, AR9590)
Bond Primary Antibody Diluent (Leica, AR9352)

Supplementary Table S1: List of qRT-PCR probes

Gene	Roche Universal Probe Library System		
	Forward Primer (5’ > 3’)	Reverse Primer (5’ > 3’)	UPL Probe
GAPDH	AGC CAC ATC GCT CAG ACA C	GCC CAA TAC GAC CAA ATC C	60
RPLP0	TCT ACA ACC CTG AAG TGC TTG AT	CAA TCT GCA GAC AGA CAC TGG	6
ACTA2	CTG TTC CAG CCA TCC TTC AT	TCA TGA TGC TGT TGT AGG TGG T	58
THY1	CCA TCC CCG GTG AAA ACT	AGG TTC ATG GTT CTG GGA TCT	25
TGFB1	ACT ACT ACG CCA AGG AGG TCA C	TGC TTG AAC TTG TCA TAG ATT TCG	31
COL1A1	GGG ATT CCC TGG ACC TAA AG	GGA ACA CCT CGC TCT CCA	67
COL1A2	GAG TCC GAG GAC CTA ATG GA	AGG GGA ACC AGG AAG ACC T	54
HAS1	GCC GGA GAG AAG AGA GAG C	GAA GGC GAT GGT CAG CAC	18
HAS2	CTC CGG GAC CAC ACA GAC	TCA GGA TAC ATA GAA ACC TCT CAC AA	73
HAS3	ACC ATC GAG ATG CTT CGA GT	CCA TGA GTC GTA CTT GTT GAG G	25
NID1	CAG TTT TCA GAT GAG GGA ACG	TGA AGG CCA GTT TCA CAG TAG TT	5
FN1	GGG AGA ATA AGC TGT ACC ATC G	TCC ATT ACC AAG ACA CAC ACA CT	25

Supplementary Table S2: Roche LightCycler480 Program

Target temperature (°C)	Acquisition mode	Hold	Ramp rate (°C/s)	Second Target (per °C)	Step size (°C)
**Pre-Incubation**					
94	None	7 min	4.8	0	0
**Amplification**					
94	None	15 sec	4.8	0	0
60	None	30 sec	2.5	50	0.5
72	Single	15 sec	1.5	0	0
**Cooling**					
40	None	30 sec	2.5	0	0

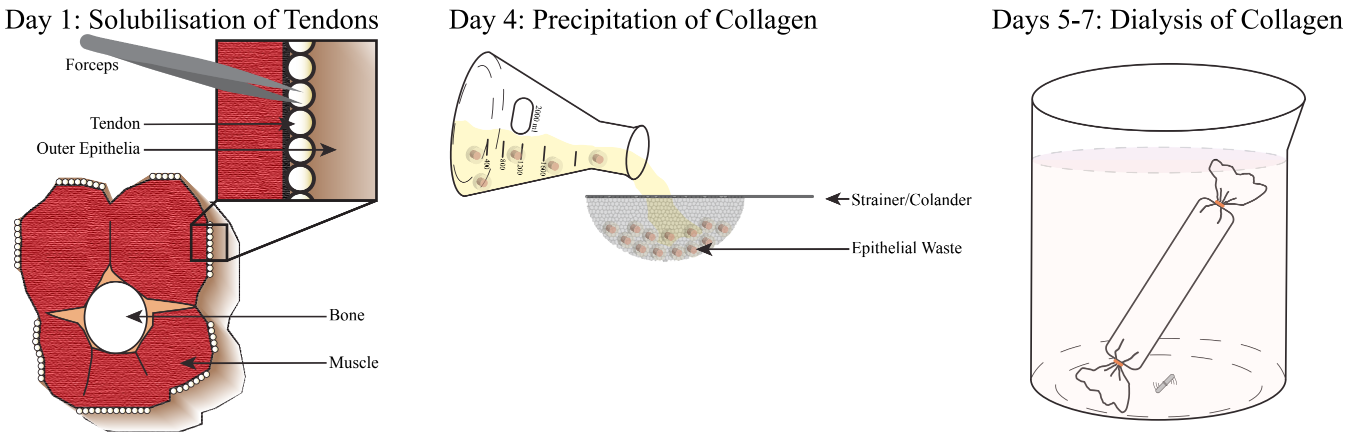

Supplementary Figure S1. Schematic representation of the key steps in the production of acid-extracted kangaroo tail collagen. Day 1, tendons from kangaroo tail are pulled out with tweezers and solubilised in 0.5 M acetic acid. Day 4, epithelial waste is filtered out of the solubilised collagen solution, prior to salt precipitation and overnight incubation in ~500 ml of 0.25 M acetic acid. Days 5-7, collagen I is dialysed against 6-8 changes of 17.4 mM acetic acid.

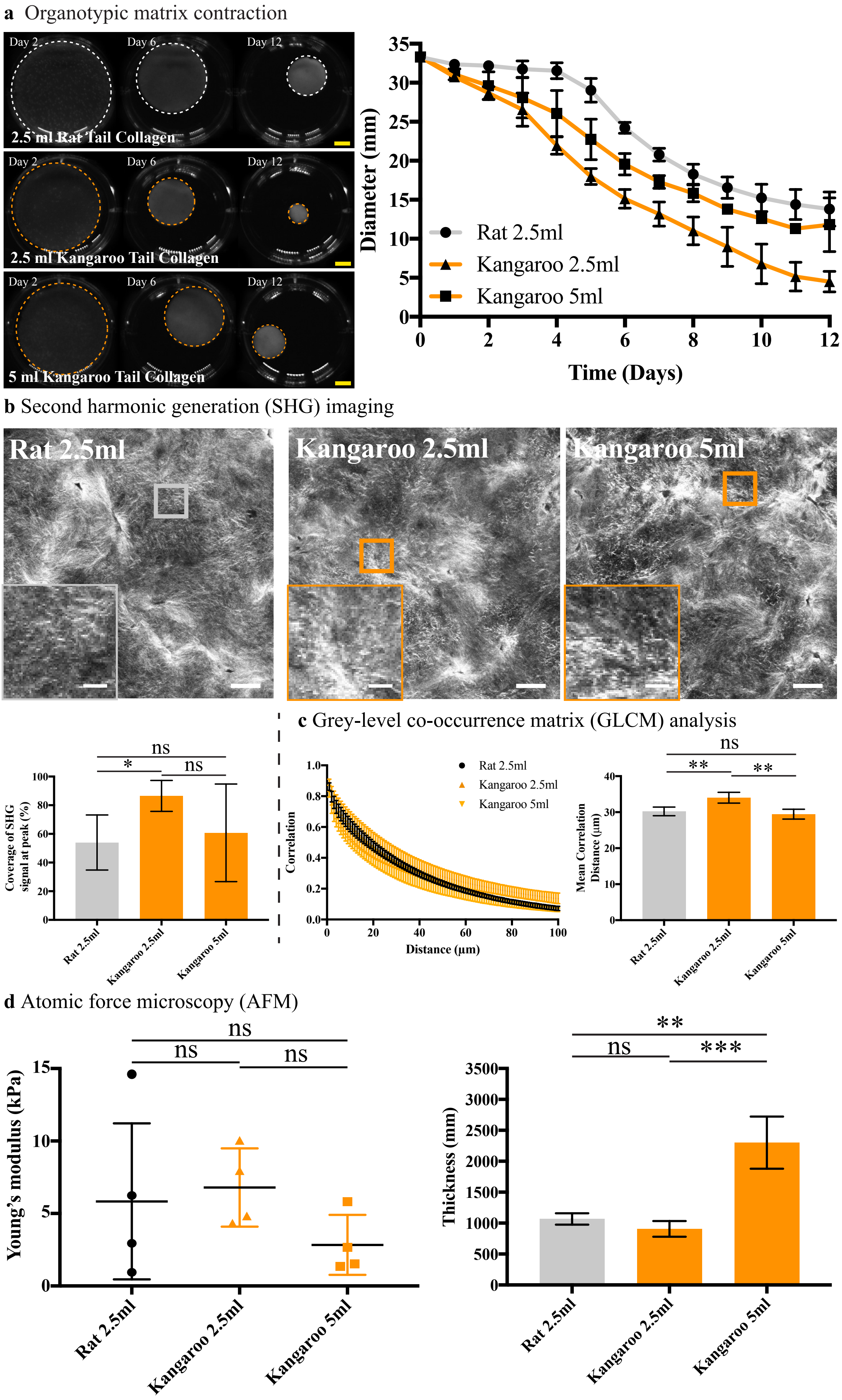

Supplementary Figure S2. Assessment of rat and kangaroo tail collagen matrix integrity and structure. (a) Fibroblast-driven matrix contraction for both rat and kangaroo tail collagens (n=4, scale bars: 5 mm). (b) Contracted matrices were then subjected to second harmonic generation (SHG) imaging of collagen crosslinking (n=4, scale bars: 50 μm, scale bars (insets): 12.5 μm), (c) grey-level co-occurrence matrix (GLCM) analysis of matrix texture (n=4) and (d) atomic force microscopy (AFM) of matrix stiffness and thickness by AFM probe engagement (n=4). Mean ± SD.

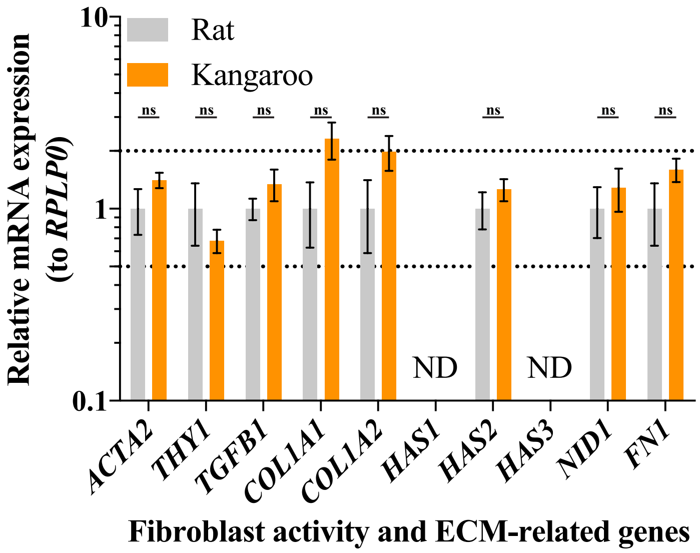

Supplementary Figure S3. Quantitative real-time PCR (qRT-PCR) analysis of relative mRNA expression, normalized to *RPLP0*, of genes indicative of fibroblast activity and matrix deposition (n=4). ND – not detected. Mean ± SEM.
